# Role of fatty acid transport protein 4 in metabolic tissues: insights into obesity and fatty liver disease

**DOI:** 10.1042/BSR20211854

**Published:** 2022-06-01

**Authors:** Huili Li, Thomas Herrmann, Jessica Seeßle, Gerhard Liebisch, Uta Merle, Wolfgang Stremmel, Walee Chamulitrat

**Affiliations:** 1 Department of Internal Medicine IV, University of Heidelberg Hospital, Im Neuenheimer Feld 410, 69120 Heidelberg, Germany; 2 Department of Gastrointestinal Surgery, Union Hospital, Tongji Medical College, Huazhong University of Science and Technology, Wuhan 430022, China; 3 Westkuesten Hospital, Esmarchstraße 50, 25746 Heide, Germany; 4 Institute of Clinical Chemistry and Laboratory Medicine, University Regensburg, Franz-Josef-Strauss-Allee 11, 93053 Regensburg, Germany

**Keywords:** acyl CoA synthetase, fatty acid transport protein 4, fatty acids, lipidomics, phospholipids, triglycerides

## Abstract

Fatty acid (FA) metabolism is a series of processes that provide structural substances, signalling molecules and energy. Ample evidence has shown that FA uptake is mediated by plasma membrane transporters including FA transport proteins (FATPs), caveolin-1, fatty-acid translocase (FAT)/CD36, and fatty-acid binding proteins. Unlike other FA transporters, the functions of FATPs have been controversial because they contain both motifs of FA transport and fatty acyl-CoA synthetase (ACS). The widely distributed FATP4 is not a direct FA transporter but plays a predominant function as an ACS. FATP4 deficiency causes ichthyosis premature syndrome in mice and humans associated with suppression of polar lipids but an increase in neutral lipids including triglycerides (TGs). Such a shift has been extensively characterized in enterocyte-, hepatocyte-, and adipocyte-specific Fatp4-deficient mice. The mutants under obese and non-obese fatty livers induced by different diets persistently show an increase in blood non-esterified free fatty acids and glycerol indicating the lipolysis of TGs. This review also focuses on FATP4 role on regulatory networks and factors that modulate FATP4 expression in metabolic tissues including intestine, liver, muscle, and adipose tissues. Metabolic disorders especially regarding blood lipids by FATP4 deficiency in different cell types are herein discussed. Our results may be applicable to not only patients with FATP4 mutations but also represent a model of dysregulated lipid homeostasis, thus providing mechanistic insights into obesity and development of fatty liver disease.

## Introduction

Dietary fats, especially fatty acids (FAs), are essential biochemicals for cellular structures and energy production. In organisms, FAs essentially participate in all cellular metabolic pathways with the requirement that they first are activated to their acyl-CoA derivatives. Acyl-CoAs participate in three main metabolic pathways including β-oxidation to generate energy, esterification to be stored as triacylglycerols/triglycerides (TGs), and the synthesis of structural lipids, such as phospholipids (PLs), sphingolipids (SPLs), plamalogens, and cholesterol esters (CEs). Other pathways include FA elongation, insertion and removal of double bonds, protein acylation, gene expression, signalling, and regulation of intracellular functions. The physiological and nutritional states dictate which direction FAs are trafficked towards [1–3]. The gut is the organ designated for FA absorption [4]. The liver is the most important organ for regulation of FA metabolism [5,6]. The muscle and adipose tissues predominantly play a role in FA oxidation and storage [7–11]. The state of homeostatic FA metabolism in these tissues and blood circulation can be seen by clinical biochemistry parameters, such as, blood lipids [12], liver fats [13,14], insulin release [15], and the state of insulin sensitivity [16–18]. Recently, metabolic (dysfunction) associated fatty liver disease (MAFLD) has been suggested by experts as a more appropriate terminology than the commonly used non-alcoholic fatty liver disease (NAFLD) [19–22], thus emphasizing the importance of metabolic homeostasis. In this context, the disturbance in FA metabolic homeostasis is known to contribute to MAFLD/NAFLD, obesity, and insulin resistance [23,24].

**Figure 1:**
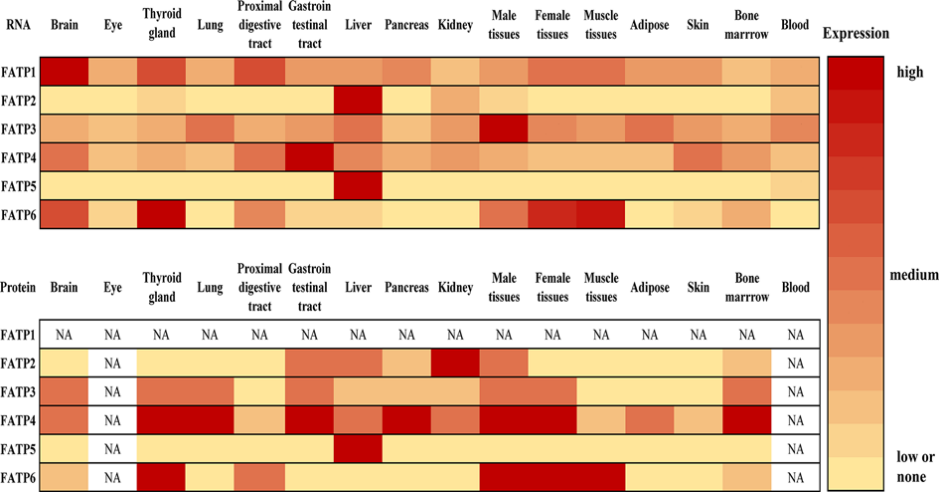
Heat map for mRNA expression patterns and protein levels of human FATPs in different organs Data are based on the Human Protein Atlas (https://www.proteinatlas.org/). NA represents no data available.

**Figure 2:**
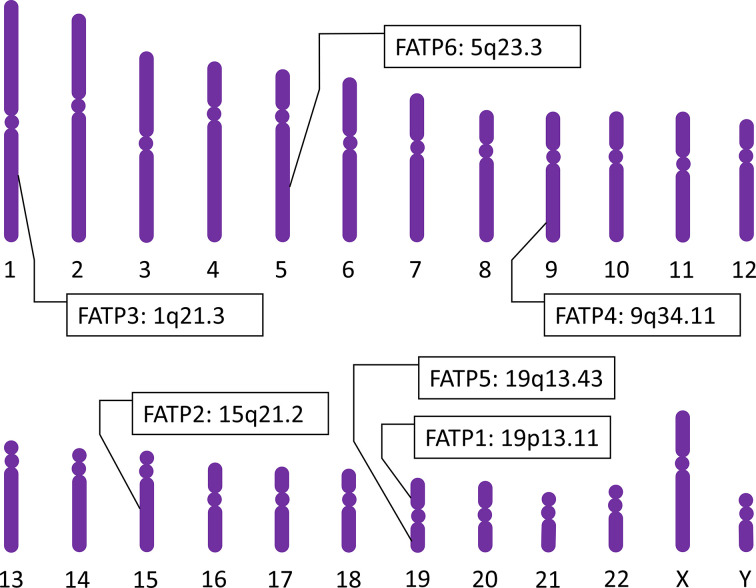
Human FATP family members FATP family genes in human chromosomes (based on chromosome painting [35–38]) mapped to 19p13.11 for FATP1 [35,36], 15q21.2 for FATP2 [35,37], 1q21.3 for FATP3 [35], 9q34.11 for FATP4 [35,38], 19q13.43 for FATP5 [35,39], and 5q23.3 for FATP6 [35,40].

In the past, the uptake of FAs by cells had been supposed to be mediated by the mechanism of FA diffusion which is based on concentration gradients of FAs [25–27]. This non-specific transport mechanism however cannot account for the high-affinity uptake and selectivity of FAs by a specific cell type [28–30]. The discovery of facilitated transport catalyzed by FA transporters, including FA translocase/CD36 (FAT/CD36) and solute carrier family 27A members, namely FA transport proteins (FATPs), seems to aid the mechanism of FA-uptake specificity [23,26,28,31]. In 1994, the first FATP was identified in adipocytes [31]. Five distinct FATPs in the mouse and six different human FATPs were subsequently reported [28]. FATPs 1–6 are diversely expressed in different organs (Figure 1, available in “Human Protein Atlas”: https://www.proteinatlas.org/ [32–34]). FATPs 1–6 genes are mapped to the chromosome 19p13.11 [35,36], 15q21.2 [35,37], 1q21.3 [35], 9q34.11 [35,38], 19q13.43 [35,39], and 5q23.3 [35,40], respectively (Figure 2, the schematic diagram of human chromosomes based on chromosome painting [41–44]).

Although there have been various studies in support of FA transport functions of FATPs [28,31,40,45–47], there has also been ample evidence revealing that these FATP members exhibit enzymatic activity of fatty acyl-CoA synthetases (ACSs) [39,45,48–55]. ACS-catalyzed thioesterification is considered the key fundamental reaction in cellular lipid metabolism including β-oxidation (and ketone bodies), FA elongation/desaturation, and the synthesis of neural lipids (TGs and CEs) and polar lipids (PLs and SPLs) [56,57]. Unlike ACS enzymes, all six mamalian FATP members exhibit activities towards not only long-chain FAs (LCFA, e.g*.*, palmitate C16:0 or oleate 18:1) but also very long-chain FAs (VLCFA) lignocerate C24:0 as a substate. Therefore, FATPs have been named as very-long-chain acyl-CoA synthetases (ACSVLs) as shown in Table 1 [57–63]. FATP1 or ACSVL4 predominantly participates in FA uptake in adipocytes and muscle [31,64,65] by its translocation from the perinuclear compartment to plasma membrane [47,66] in response to a hormone, such as, insulin [47,67–69] or isoproterenol [70]. Metabolic labelling studies on ACS activity using [^3^H]palmitate or [^3^H]lignocerate indicates that FATP1 has no preference for either substrate [49,50]. FATP2 or ACSVL1 has two splice variants FATP2a and FATP2b, and both of which are able to mediate FA uptake, but only FATP2a exhibits ACS activity [71]. FATP2 is primarily expressed in liver and kidney and is found in both microsomes and peroxisomes [57,72]. Liver-specific Fatp2 knockdown in mice via adenovirus-based short hairpin RNA vectors is shown to suppress hepatic steatosis and improve insulin sensitivity indicating significant role of FATP2 in hepatic FA uptake [72]. FATP3 or ACSVL3 is shown to catalyze FA activation but not FA transport in endothelial cells [51]; however, it is a target of vascular endothelial growth factor B to mediate an indirect FA uptake [73]. Unlike other FATPs, FATP4 or ACSVL5 is widely expressed in brain, liver, adipose tissues, skeletal muscle, heart, and intestine [57,63]. FATP4 is localized mainly at the endoplasmic reticulum (ER) and partially localized to peroxisomes [55,60]. It plays a role in FA transport via ACS-activated vectorial acylation [48,55,60–63], that the depleted intracellular FA concentrations indirectly trigger an FA uptake. Metabolic labelling ACS activity assays show that FATP4 overexpression increases the activity by 2 folds with palmitate and 5 folds with lignocerate indicating that the FATP4 gene encodes an ACS with substrate specificity towards VLCFAs [62,63]. Remarkably, FATP4 mutations or deletion cause ichthyosis premature syndrome (IPS) and lethality of neonates in humans [74,75] and mice [76–79]. An uptake/flux study using ^14^C-radiolabeled oleic acid has demonstrated that FATP5 or ACSVL6 which is localized on the plasma membrane, functions as an FA transporter in the hepatocytes [80]. Consistently, FATP5 silencing *in vivo* attenuates diet-induced NAFLD [81]. Lastly, FATP6 or ACSVL6 is shown to be an FA transporter and is reported only in human heart [40]. Taken together, FATP enzymes play a povital role in FA uptake and metabolism based on their diverse expression and cellular localization in different cell types. Thus, altered expression of FATPs in metabolic tissues have been recognized to play a major role in diet-induced obesity, hepatic steatosis, insulin resistance, and metabolic syndrome diseases in mice [64,65,68,72,81] and humans [23,24,38,82,83].

**Table 1:** Names of FATP/ACSVL family members

Official gene symbol	Name as fatty acid transporter	Name as very-long-chain acyl-CoA synthetase^*^	Previous/other names
SLC27A1	FATP1	ACSVL4 (ACSVL5)^#^	FATP
SLC27A2	FATP2	ACSVL1	VLCS, VLACS, VLACS1
SLC27A3	FATP3	ACSVL3	VLCS-H3, VLACS3
SLC27A4	FATP4	ACSVL5 (ACSVL4)^#^	------
SLC27A5	FATP5	ACSVL6	VLCS-H2, VLACS-R, BACS
SLC27A6	FATP6	ACSVL2	VLCS-H1, VLACS2

* Name as very long-chain acyl-CoA synthetase for the FATP/ACSVL family members are based on the nomenclature system adopted for the long-chain acyl-CoA synthetase (VLCS) family [57–61].

# In some literatures, FATP4 was named as ACSVL4 and FATP1 was named as ACSVL5 [61–63].

In the overall lipid metabolism, ACS or FATP enzymes also play a significant role in FA degradation pathway via α- and β-oxidation. It is generally accepted that β-oxidation of LCFAs and VLCFAs occurs primarily in mitochondria and peroxisomes, respectively. By using [^14^C]oleate in β-oxidation metabolic labelling assay, it is demonstrated that hepatic lon-chain fatty ACS1 (ACSL1) is important for mitochondrial β-oxidation of LCFAs [84], Moreover, ACSL1-mediated β-oxidation in adipocytes is required in cold thermogenesis [85]. It is also reported that ACSL3 mediates β-oxidation of FAs in lung cancer cells [86]. For FATP family, FATP1, which is localized in mitochondria of mouse skeletal muscle, is shown to increase β-oxidation resulting in hyperketonemia *in vivo* [87]. Interests have been especially paid on β-oxidation of VLCFAs in peroxisomes and the identification of the responsible ACS/FATP enzymes. This is because the impairment of VLCFA degradation leads to abnormal accumulation of VLCFAs in tissues and plasma in patients with the neurodegenerative disease X-linked adrenoleukodystrophy (X-ALD) [56,88]. So far, ACSL4, FATP2, and FATP4 have been identified to be present in peroxisomes [56]. ACSL4 may have a specialized role in peroxisomes since it has specificity for activation of polyunsaturated fatty acids (PUFAs). While FATP2 activates both palmitate and lignocerate [57], this enzyme is a major contributor to peroxisomal ACSVL activity [72]. However, FATP2 deletion in mice does not lead to accumulation of VLCFAs in livers and kidneys, although VLCFA β-oxidation rates are reduced [88]. Likewise, fibroblasts from X-ALD patients show decreased perosixomal ACSVL activity; however, FATP2 expression is not significantly different between normal controls and X-ALD [89]. Thus, it appears that FATP2 does not have a direct role in X-ALD pathology. Metabolic labelling study on β-oxidation by the conversion of [1-^14^C]FA to ^14^CO_2_ has demonstrated the specificity of FATP4 for VLCFA lignocerate [55]. Consistent with VLCFA preference and localization to peroxisomes, it has been shown that FATP4 interacts with fatty acid synthetase and the peroxisomal ATP-binding cassette half-transporter, adrenoleukodystrophy protein (ALDP) whereby its mutations are the primary cause of X-ALD [90]. Mutations of ALDP however does not alter its interaction with FATP4. It is postulated that FATP4 localized at the cytoplasmic side of the peroxisomal membrane activates VLCFAs to CoAs which are transported by ALDP for β-oxidation in the peroxisomal lumen. Furthermore, it is shown that VLCFA-CoAs catalyzed by FATP4 can undergo elongation and these ultraVLCFA-CoAs are subsequently incorporated into complex lipids [91]. Thus, FATP4 paticipates in X-ALD pathology by elevating the levels of lipids containing VLCFAs and ultraVLCFAs in the cytosol which are exported extracellularly.

Among human FATP family members, FATP4 is broadly distributed and highly expressed in metabolically active tissues such as adipocytes [23,31,61,67,69] and adipose tissues of obese individuals [38,82,83]. Although the protein level of FATP4 is high in thyroid glands (Figure 1), there has been no investigations on FATP4 in these tissues. FATP4 gene is evolutionally conserved and is the only FATP known so far to cause severe neonatal abnormalities or death as reported in FATP4-null mice [76–79], as well as IPS patients with FATP4 mutations seen mainly in Europe [74,75,92–96] and some in Asia [97,98]. The deletion of Fatp4 in mice or mutations of FATP4 in humans lead to wrinkle-free restrictive dermopathy with phenotypes including ichthyosis, embryo/neonatal lethality, and death shortly after birth. Interestingly, a targeted disruption in the upstream of exon 2 of Fatp4 gene [78] results in neonatal death at earlier time than the disruption of exon 3 [76]. This indicates a different truncated form of Fatp4 in mice. Furthermore, FATP4 deletion in mice leads to epidermal hyperproliferation [99], and these mutants also represent a model of congenital ichthyosis [100]. Of note, epidermis-specific inactivation of Fatp4 in mice does not lead to death but causes milder hyperkeratosis when compared with the phenotypes of Fatp4-null mice [99]. Speculatively, the severe phenotypes seen in Fatp4-null mice could be due to the deficiency in immune cells such as macrophages that might lead to inflammatory activation. In support of this notion, many patients with FATP4 mutations survive neonatal abnormalities, and adult patients show signs of not only hyperproliferative hyperkeratosis but also allergies and eosinophilia [92,101]. It is surmised that these patients may exhibit high blood lipids and insulin resistance [38,82,83] due to the mutations in metabolically active tissues, such as, adipose tissues and liver. In the past two decades, research efforts have been made to investigate FATP4 role in the skin of mice and humans. Key findings on Fatp4-null mice include a decrease in ceramides containing ultraVLCFAs [76,79,102] including omega-O-acylceramides [79,102] and omega-hydroxyceramides [102]. The latter two constitute the major compounds of the corneocyte lipid envelope which is required for normal permeability barrier in skin. Concomitant with decreased ultraVLCFA-ceramides, there is an accumulation of neutral lipids as reported in skin of IPS patients [75] and Fatp4-null mice [76,77]. As discussed in this review, more recent studies in our laboratories using tissue-specific knockout (KO) mice have suggested FATP4 functions on FA metabolism in metabolic tissues including the intestine, liver, and adipose tissues. We also have reviewed other published studies to discuss the relationships between FATP4 with obesity and fatty liver disease.

## General characteristics of FATP4

Herrmann and co-workers [63] have stated that the murine Fatp4 gene contains 1929 bp in the open-reading frame encoding a polypeptide of 643 amino acids, and is assigned to mouse chromosome 2 band B (syntenic to the region 9q34 encompassing the human gene). In 2004, Gertow and co-workers [38] have stated that the human FATP4 gene contains 12-coding exons spanning more than 17 kb of genomic DNA and maps to chromosome 9q34. In 2007, Watkins and co-workers [35] have shown that the human FATP4 gene contains 13 exons and mapped the FATP4 gene to the plus strand of chromosome 9q34.11 by genomic sequence analysis (Figure 2).

The FATP family is conserved from invertebrates to vertebrates [35]. FATP1 and FATP4 in vertebrates are clustered with FATP genes in invertebrates. FATP2, FATP3, FATP5, and FATP6 are present only in vertebrates among which FATP6 being present only in humans [103]. FATP1 and FATP4 are broadly distributed, and they could similarly exhibit the evolutionary conserved functions [103]. Within the coding region, the exon-intron structures of the murine Fatp4 gene are identical to its human counterparts and human FATP1 gene is closely resembled to the mouse gene [63]. Thus, FATP1 and FATP4 may play a complementary role because it has been shown that Fatp1 can compensate for Fatp4 in the developing mouse epidermis [104–106]. It is known that FATP1 is highly expressed in hormone-sensitive tissues including white adipose tissue and soleus muscle, and that a hormone insulin is necessary for FATP1 to mediate FA uptake [64,65]. Being different from FATP1, FATP4 indirectly mediates FA uptake in not only insulin-sensitive [61] but also insulin-insensitive tissues [55,60,62,63]. In adipocytes, the knockdown of FATP1 or FATP4 could decrease cellular TG levels by inducing TG lipolysis [67]. This knockdown also leads to an increase in the uptake of 2-deoxyglucose. As glucose is converted to acetyl-CoA and used for syntheses of fatty acids and subsequently TGs, hence FATP1 or FATP4 knockdown may replenish the decreased TGs by enhancing glucose uptake. It is thought that FATP1 and FATP4 may have overlapping biological functions, and that the increased FATP1 expression may reduce atopic abnormalities and death in surviving patients with FATP4 mutations [106].

In humans, a G/A polymorphism within exon3 of FATP4 that gives rise to a Gly209Ser substitution is shown to be associated with insulin resistance [38]. The heterozygous carriers of the Ser209 allele show improved metabolic parameters, such as, lower body-mass-index, blood TGs and lipoprotein levels, systolic blood pressure, and insulin concentrations. Hence, FATP4 has been proposed as a key and multifunctional FATP enzyme to be involved in lipid metabolism that leads to human obesity and insulin resistance [38,82,83].

## FATP4 in metabolic organs and tissues

### FATP4 in the gut

#### Expression pattern of FATP4 in the gut

FATP4 is highly expressed in enterocytes or intestinal mucosa reported in many vertebrates including yellow catfish [107,108], chickens [109,110], mice [46], rats [111], pigs [112–114], and humans [115]. Interestingly, no significant differences in FATP4 expression are observed between duodenum, jejunum, ileum, proximal colon, and distal colon in human gut [115]. According to the Human Protein Atlas (https://www.proteinatlas.org/), FATP4 expression is relatively lower in colon than other segments of the gut. Nonetheless, FATP4 expression in intestine is higher than in any other organs (Figure 1), and therefore FATP4 in this tissue has been intensively investigated in the past two decades.

#### FATP4 role on lipid absorption and metabolism in the gut

After the discovery of FATPs during 1994–1998 [28,31], FATP4 was proposed to be a major importer of dietary FAs because of its predominant expression in gut [46,115,116]. Overexpression of FATP4 in cells (HEK293 and primary mouse enterocytes) increases long-chain FA uptake, while FATP4 deficiency inhibits intestinal uptake [46]. At the time, these results suggested that FATP4 could be a principal FA transporter in enterocytes, and that FATP4 inhibitors might be of therapeutic use as anti-obesity drugs. Although mice that are heterozygous for the exon 2 Fatp4 mutation were viable and they showed marked reduction of FA uptake in enterocytes, however they did not show any defects in fat absorption [78]. This finding in 2003 was the first report to undermine the importance of FATP4 in intestinal FA uptake. During 2001–2007, FATP4 was reported to exhibit ACS activities with specificity towards VLCFAs [55,58,62,63]. Thus, FATP4 is thought to be bifunctional as an FA transporter and ACS, and that FATP4-dependent vectorial FA acylation works in concert to mediate FA influx [48,55,60–63], which is the same mechanism as FATP1 [49,50]. The lack of effects on intestinal FA absorption in Fatp4-allele deleted heterozygous mice [78] may also suggest some subtle uncharactereized metabolic changes driven by global FATP4 deletion.

During 2006–2014, more detailed molecular studies have confirmed the ER localization of FATP4 in cultured cells [60,117,118]. It is shown that FATP4 works synergistically with the plasma membrane translocase FAT/CD36 in facilitating FA transport via ACS-driven mechanism [117]. Another study has demonstrated that the rescue of Fatp4-null mice with keratinocyte-specific FATP4 overexpression requires an intact ACS domain [119]. As FATP4 overexpression increases ACS activity in hepatoma cells [120], thus the intact ACS domain is necessary for the functions of FATP4 under *in vitro* and *in vivo* conditions. While FATP4 exhibits ACS-specific activities with the preference towards VLCFA C24:0 *in vitro* [60,62], the skin and intestine of Fatp4-null mice also show reduced esterification of C24:0, but not C16:0 or C18:1 [63]. The defects in esterification of VLCFAs to ceramides could be observed in skin Fatp4-null mice, and this event underlies the observed skin abnormalities [76,79,102]. Because Fatp4-allele deleted heterozygous mice did not show any defect in intestinal fat absorption [78], we surmise that Fatp4-dependent ACS activities towards the abundant LCFAs may not lead to an FA uptake, but rather to other metabolic pathways. Taken together, FATP4 indeed acts as an ACS and is not essential for FA absorption in the intestine, and this notion was discussed in 2018 by Cifarelli and Abumrad [121] as well as Schreck and Melzig [122].

In 2009, keratinocyte-specific Fatp4-rescued Fatp4-null mice [119] were used to investigate the effects of FATP4 on lipid absorption after feeding with a Western diet [123]. With an exception for keratinocytes, these mutants would still exhibit Fatp4 deletion in all other cell types, such as, adipocytes, immune cells, muscle cells, hepatocytes, and enterocytes. Results showed no difference in food consumption, growth, and body-weight gain. No intestinal TG absorption and fecal fat losses could be found. Thus, FATP4 is dispensable for intestinal lipid absorption in mice. No difference in hepatic lipid contents and TG secretion could be observed after injection with a lipoprotein lipase inhibitor Tyloxapol. This indicates no FATP4 role on hepatic FA uptake as well. This lack of FATP4 role on lipid absorption is supported by an earlier study in 2006 demonstrating a failure of FATP4-specific inhibitors to attenuate fat absorption *in vivo* [124]. Despite no difference in intestinal TG absorption kinetics, these mutants fed Western diet showed a significant increase in enterocyte TG and FA contents. This suggests that FATP4 deficiency may shift FA activation towards TG synthesis in intestine. This notion can be supported by other evidence for an increase of neutral lipids including TGs in the skin of IPS patients with FATP4 mutations [75] and Fatp4-null mice [76,79]. Although FATP4 is considered an important protein in lipid absorption research [125], FATP4 deficiency does not per se affect FA uptake, but it may induce a shift of FA metabolism to TGs. Besides intestine, the metabolic changes in other tissues, such as muscle and adipose tissues, may contribute to the lack of effects on blood TG levels in these mutants fed Western diet [123]. Nonetheless, the determination of blood chylomicrons under this condition would have been useful to specifically clarify intestinal fat absorption.

Similar to Fatp4, LCFA ACS-5 (or Acsl5 in the mouse) is also highly expressed in mouse jejunum [126]. While Acsl5 KO mice fed high-fat diet (HFD) displayed a decrease in ACS activity, they did not show any changes in dietary FA absorption and body-weight gain. It is concluded that the residual ACS activity in Acsl5 KO mice is sufficient to maintain normal levels of FA absorption. These results are similar to those from keratinocyte-rescued Fatp4-mutants fed Western diet [123] indicating that only minimal levels of intestinal ACS/FATP enzymes are nescessary for normal FA absorption.

**Figure 3:**
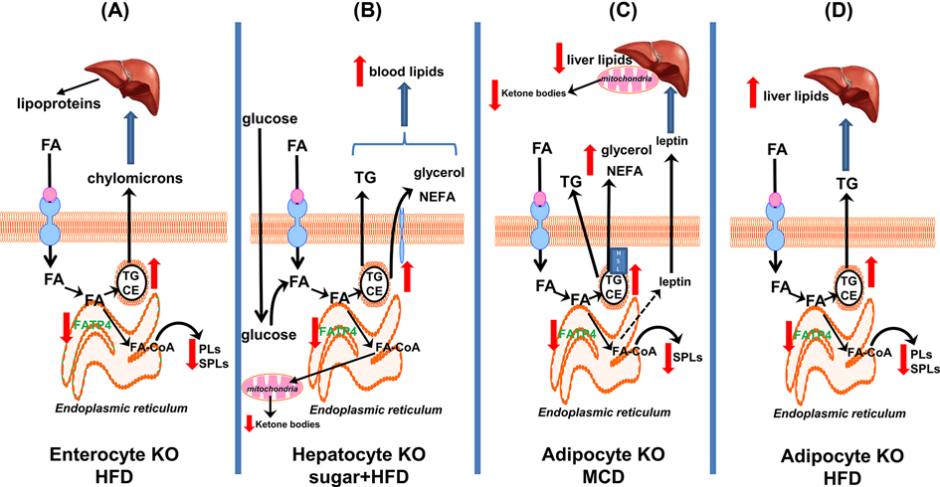
Mechanistic scheme of lipid metabolism in tissue-specific Fatp4-deficient mice FAs enter cells via a number of plasma membrane transporters. (**A**) Fatp4 deficiency in enterocytes under HFD feeding suppresses FA activation and synthesis of PLs and SPLs. This allows accumulation of TGs which are exported as chylomicrons that are taken up by liver and subsequently released as lipoproteins. (**B**) Fatp4 deficiency in hepatocytes under sugar+HFD feeding also leads to FA shift to TGs which are readily hydrolyzed to glycerol and NEFA resulting in an increase in blood lipids. (**C**) Fatp4 deficiency in adipocytes under MCD diet feeding leads a decrease in adipocyte SPLs and an increase in TGs which are hydrolyzed by HSL to glycerol and NEFA. Despite increased blood lipids, Fatp4 deficiency leads to enhanced release of leptin which may protect mice from fatty liver in this model. (**D**) Fatp4 deficiency in adipocytes under HFD feeding leads to a decrease in PLs and SPLs, an accumulation of adipocyte TGs, and fatty liver development. Abbreviations: CE, cholesterol ester; FA, fatty acids; FA-CoA, fatty acyl CoA; FATP4, fatty acid transport protein 4; HFD, high-fat diet; HSL, hormone-sensitive lipase; MCD diet, methionine-choline deficient diet; NEFA, non-esterified free fatty acids; PL, phospholipid; SPL, sphingolipid; TG, triglyceride.

Intestine-specific Fatp4-deficient mice have been generated in our laboratory and our initial report was published in an abstract form [127]. Under normal diet, these Fatp4-deficient mutants showed a decrease in intestinal PLs, sphingomyelin, and ceramides concomitant with an increase in intestinal TGs and CEs. Upon starvation, these mutants showed a moderate increase in blood lipoproteins. Upon feeding with a high-fat high-cholesterol diet, these mutants showed a strong increase in blood lipids and lipoproteins. As shown in Figure 3A, rather than the anticipated inhibition of lipid absorption, intestinal Fatp4 deletion appears to worsen metabolic conditions as seen in elevation of blood lipids and lipoproteins. We propose a shift of FA activation towards TGs in FATP4-deficient intestine, and this event leads to the secretion of chylomycrons by intestine and subsequent secretion of lipoproteins by liver. Similarly to keratinocyte-rescued Fatp4-null mutants [123], an increase in blood lipoproteins including chylomicrons in our intestine-specific Fatp4-deficient mice is not sufficient to elevate hepatic steatosis under dietary stress [127] (Figure 3A).

#### The transcriptional factors affecting FATP4 transcription in the gut

A number studies have investigated intestinal expression of FATP4 *in vivo*, and these studies may provide some clues regarding the regulation of FATP4 in the gut. Intestinal FATP4 expression is not affected by gene deletion of a mucin-generating enzyme [128] or FA uptake enzyme FAT/CD36 [129]. However, FATP4 is down-regulated in the intestine of mice with global deletion of lysophosphatidylcholine acyltransferase3 (LPCAT3) [130] or group VIA calcium-independent phospholipase A2 (iPLA2β) [131]. LPCAT3 catalyzes the acylation of lysophosphatidylcholine (LPC) to phosphatidylcholine (PC). On the opposite, iPLA2β hydrolyzes PLs to lysoPLs at sn-2 position, and it has a greater specificity for hydrolyzing phosphatidylethanolamine (PE) compared with other PLs. This implies that the deletion of either of these genes may lead to changes in intestinal PC and PE that subsequently affect FATP4 transcription and expression. Specifically, it appears that a decrease in intestinal PC/PE ratio by LPCAT3 or iPLA2β deficiency would result in suppressed FATP4 expression. The mechanism for this may involve attenuated activation of an ER transmembrane protein inositol-requiring enzyme1α leading to suppressed transcription of homeostatic genes including FATP4 [131]. Upon inhibiting intestinal cholesterol absorption either by the deletion of Niemann-Pick type C-1 like protein 1 or treatment with a lipid-lowering drug Ezetimibe [132], intestinal FATP4 expression is shown to be suppressed. This suggests a link between FATP4 and cholesterol metabolism in intestine. Fasting for 1–13 days is also shown to down-regulate intestinal FATP4 expression which can be reversed upon refeeding [133]. While short-term 4-day HFD feeding down-regulates intestinal FATP4 expression [134], chronic feeding with fructose [135], HFD [136] or oil [137] however does not alter intestinal FATP4 expression. Thus, intestinal FATP4 appears to be regulated by changes in PC/PE ratio and cholesterol metabolism as well as acute changes of gut nutrients due to starvation and fat exposure time.

**Figure 4:**
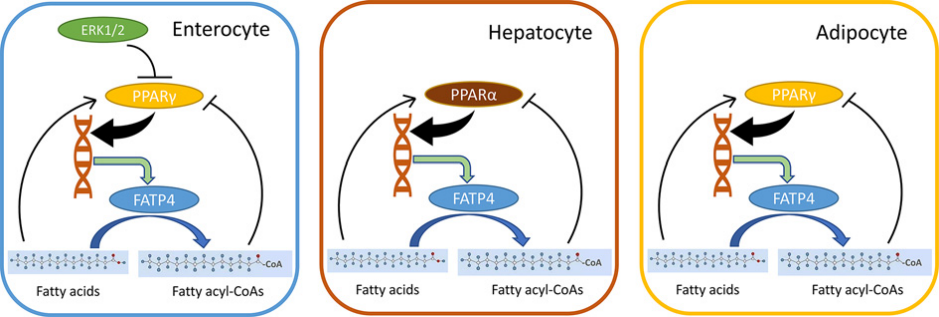
Feedback loop regulation of FATP4 by its substrates/products and PPARs Feedback loops among PPARs, FATP4, FAs, and fatty acyl-CoAs in the enterocyte, hepatocyte, and adipocyte. The transcription of FATP4 is activated by PPARγ in enterocytes and adipocytes, and by PPARα in hepatocytes. As FAs activate the transcriptional activities of PPARs, fatty acyl-CoAs activated by FATP4 in turn inhibit PPARγ and PPARα in the corresponding cells. In addition to fatty acyl-CoAs, ERK1/2 also inhibits PPARγ in enterocytes.

We now discuss transcriptional factors that regulate intestinal FATP4 transcription. It has been shown that peroxisome-proliferator-response element is present in the upstream region of FATP gene, and proxisome proliferator-activated receptor (PPAR) agonists such as linoleic acids and PUFAs may transcriptionally regulate FATP4 expression [138]. It has also been shown that the activation of mitogen-activated protein kinase member ERK1/2 induces an inhibition of PPARγ resulting in down-regulation of FATP4 expression in piglet intestine [139]. Thus, ERK1/2 and PPARγ are upstream regulators of FATP4 (Figure 4). It is reported that intestinal Fatp4 expression is not altered by an administration of a PPARα agonist in mice [140]. Thus, PPARγ is a key regulator of intestinal FATP4. Moreover, it is shown that fatty acyl-CoAs are able to suppress PPARγ activation and thus they play an opposing role from FAs [141]. Hence, intestinal FATP4 and its substrate FA and product fatty acyl-CoAs may regulate PPARγ in a feedback-loop manner (Figure 4). Taken together, under dietary stress FATP4 inactivation may increase FAs which are incorporated into intestinal TGs [123] and released as chylomicrons and subsequently lipoproteins [127] (Figure 3A).

### FATP4 in the liver

#### FATP4 in fatty liver disease

In mammals, the liver plays an essential role in maintaining metabolic homeostasis of lipids [24]. Compared with FATP2 and FATP5, FATP4 is a minor FATP in the liver [28,31,116]. However, it is shown that FATP4 protein expression is increased in fatty livers in genetic *ob/ob* and HFD-fed mice [142–144]. Since accumulation of hepatic lipids is a common consequence of obesity [145,146], current data support the notion that FA activation by FATP4 may lead to enhanced FA uptake and subsequent metabolism could lead to lipid accumulation in hepatocytes. Similar to fatty liver, FATP4 expression is also increased in adipose tissues of obese women [38,82,83,147] suggesting that TG-FA recycling and mobilization from adipose tissues via TG lipolysis may additionally exacerbate fatty liver development [148–150]. As food deprivation in mice is known to induce lipid mobilization from adipose tissues to liver, hepatic steatosis is developed under this condition together with increased hepatic Fatp4 expression [143]. This is in contrast with the reported decrease of intestinal FATP4 expression during fasting [133]. In addition to liver TGs, other deleterious lipid species have been reported to be increased in fatty liver including diacylglycerol and CEs together with a decrease in hepatic PC/PE ratio [145]. Thus, FATP4-mediated FA activation could lead to alterations of various polar lipids and neutral lipids which in turn could play a crucial role for development of MAFLD/NAFLD *in vivo*.

Mechanistically, FATP4 is localized in the ER and mitochondria of hepatocytes [120,142], which is consistent with the findings in the intestine [115] and other cell types [55,60,61,117,118]. This indicates that FATP4 in the hepatocytes could as well function as an ACS rather than a direct FA transporter. As previously reported in skin fibroblasts [55], fatty acyl-CoAs activated by FATP4 may undergo β-oxidation to ATP and ketone bodies in hepatocytes. By overexpressing FATP4 in human hepatoma cells, a significant increase in ACS activities, FA uptake, cellular lipids, and apoptosis, have been observed [120,142,151]. Collectively, FATP4 functions as an ACS in hepatocytes and its overexpression may contribute to fatty liver disease.

Although forced expression of FATP4 in hepatoma cells leads to an increase in cellular uptake and steatosis [120,142], this model may be applicable to cancer cells and may not be realistic in normal liver in which hepatic FATP4 expression is relatively low [28,31,68,116,138]. The observed high expression of FATP4 in livers of obese mice [142,151] may not per se be a direct cause of fatty liver because FATP4 can be up-regulated by dietary essential FAs known as PPAR agonists [138] (Figure 4). The first clue for no effects of Fatp4 deficiency on hepatic steatosis was already shown in keratinocyte-rescued Fatp4-null mice [123]. These mutants were treated with lipoprotein lipase inhibitor Tyloxapol which results in extremely high blood TGs, and these high levels could mask the much lower levels of metabolically generated TGs by hepatic Fatp4 deficiency. To dissect the functions of hepatic FATP4, mice with hepatocyte-specific Fatp4 deletion were generated in our laboratory.

We published our results in 2019 and demonstrated that hepatocyte-specific FATP4-deficient (Fatp4L**^-/-^**) mice under chow diet display no phenotypes regarding body weights and blood lipids [152]. Upon feeding with either a high-sugar or high-fat/high-sugar diet, Fatp4L**^-/-^** mice showed elevated levels of plasma TGs, non-esterifed free fatty acids (NEFA), and glycerol (Figure 3B). By combining body and liver weights of female and male mice, Fatp4L**^-/-^** mice under a high-sugar diet had lower weights, but they were not protected from diet-induced fatty liver. Hence, our data on liver lipids and steatosis are consistent with those from keratinocyte-rescued mutants fed Western diet [123]. Remarkably, an increase in plasma lipids of Fatp4L**^-/-^** mice fed the diets indicates worsen metabolic hepatic dysfunctions and hyperlipidemia (Figure 3B).

Since the liver plays an important role in the regulation of blood lipids [24], several FATPs in liver may inherently be involved in this process via FA uptake, FA metabolism, and subsequent secretion of lipids and lipoproteins. Although FATP2 [71,72,120] and FATP5 [80,81,153,154] are recognized as the major FATPs and FA transporters in the liver, hepatic FATP4 appears to be bifunctional, i.e*.*, mediating FA uptake when overexpressed *in vitro* [120,142] and regulating FA activation and metabolism upon its deletion *in vivo* [152]. In either condition, FATP4 is involved in the secretion of lipids. The increase in plasma NEFA and glycerol in our mutants fed high-sugar diets suggests an increase in lipolysis activities since the liver contains several intracellular lipases and hepatic lipase at plasma membrane. To investigate this possibility, we generated a stable FATP4-knockdown system in HepG2 cells and published our first results in an abstract form [155]. We observed that lipolysis lipids NEFA, glycerol, and lipoproteins were indeed increased upon FATP4 knockdown, and such increase was further exacerbated by oleate treatment. Thus, TG lipolysis mechanism would be consistent with an increase in blood lipids in Fatp4L**^-/-^** mice fed with high sugar and fat diets (Figure 3B). Apparently, TG lipolysis activities are not as efficient in the intestine and skin. This would result in an accumulation in TGs in intestine of keratinocyte-rescued Fatp4-null mice fed Western diet [123] and intestine-specific Fatp4-deficient mice [127], as well as an increase in neutral lipids in the skin of Fatp4-null mice [76,79] and IPS patients [75].

#### The transcriptional factors affecting FATP4 transcription in the liver

Hepatic FATP4 expression is increased in mouse fatty liver upon feeding with HFD [142,144] or methionine-choline deficient (MCD) [156] diet. The regulation of FATP4 in liver is different from that in intestine whereby lipid metabolism in liver appears to be more robust [5,6]. Regarding FATP4 metabolism, FATP4 deficiency may also lead to changes in FA/fatty acyl-CoA ratio that could alter the activities of PPAR transcription factors in the liver [141,157–159]. Compared with other PPARs, PPARα plays a major role on gene expression in liver [160]. PPARα is shown to up-regulate the transcription of FATP4 and multiple genes which are responsible for lipid metabolism in HepG2 cells and human liver [161,162]. Transcription factors SREBP-1c and NF-κB do not have any effects on FATP4 expression, while they can up-regulate FATP2 and FATP5 expression as well as long-chain FA uptake in the liver [163]. Thus, FATP4 expression may be predominantly regulated by PPARα and PPARγ in the liver and intestine, respectively (Figure 4). As FAs can modulate FATP4 expression [108,142,143,159], a feedback loop between PPARα and FATP4 may be operative via FAs and fatty acyl-CoAs [141,157,158] (Figure 4). This feedback loop is to some extent auto-regulated to maintain hepatic FA homeostasis by FATP4.

We further discuss the effects of FAs on hepatic FATP4. An initial palmitate or oleate exposure is shown to decrease FATP4 expression [108], and this is thought to be a protective response in preventing FA-overload and toxicity. As FA exposure persists, FATP4 expression in enterocytes is shown to be recovered to basal levels [136]; however, the recovery of FATP4 expression in hepatocytes is found to be greater than basal levels [108,142,143]. We thus propose that FATP4 in normal liver may exhibit a protective function while excessive FATP4 combined with dietary stress is cytotoxic to hepatocytes [142]. Upon FATP4 deletion in hepatocytes under high-sugar and high-fat conditions [152], homeostatic FA metabolism is then shifted towards TGs which are readily hydrolyzed to glycerol and NEFA (Figure 3B). These blood lipids may in turn lead to toxicity in peripheral tissues leading to metabolic syndromes without any increase in hepatic steatosis, which is known as non-obese MAFLD/NAFLD. The proposed mechanism in Figure 3B indeed supports an association of FATP4 polymorphisms with blood lipids and insulin resistance in humans [38,82,83].

### FATP4 in the muscle

#### FATP4 role on lipid metabolism in the muscle

Muscle is an important organ in FA metabolism and closely associated with insulin resistance [23,164]. FATP4 is expressed abundantly on the sarcolemma in skeletal muscle [165]. Other reports have shown intracellular FATP4 localization but not at the sarcolemma [118,166]. Similar to FATP4 in the intestine [60] and liver [120,142,152], FATP4 in muscle exhibits high ACS and FA-uptake activities, which are not modulated by insulin [118]. It has been shown that Fatp4, Fat/Cd36, Fatp1, and plasmalemmal FA-binding protein (pm-Fabp) mediate the transport of FAs and also increase β-oxidation in skeletal muscle of rats [167]. None of these four proteins is able to alter the rates of FA esterification into TGs in muscle. Thus, FATP4 catalyzes FAs into fatty acyl-CoAs which are directed to β-oxidation rather than esterification in the muscle.

In humans, skeletal muscle FATP4 protein is increased by ∼30%, while FATP1 protein is reduced by ∼20%, after an 8-week supervised aerobic training [168]. This supports the notion that FATP4 is involved in training-induced FA β-oxidation in skeletal muscle. Compared with FATP1, FATP4 appears to be more specific for channeling of FAs towards β-oxidation. A member of a subfamily of serine/threonine kinase Akt2 is shown to be involved in insulin- and contraction-induced FA transport in mouse muscle [169]. Akt2 is also shown to mediate intracellular retention of Fatp4 and pm-Fabp as well as the translocation of Fat/Cd36 and Fatp1 in mouse muscle. Thus, unlike FAT/CD36 and FATP1, FATP4 in the sarcolemma of muscle plays a predominant role in activating FAs for β-oxidation and energy production.

#### Factors that regulate muscle FATP4 expression

Unlike other metabolically active tissues, obesity and fat intake [170,171] as well as PPARγ activation [172] have little effects on FATP expression in muscle. This is because the muscle expresses very low levels of PPARα and PPARγ [173]. Although PPARα is reported to play a predominant role in skeletal muscle when treated with a PPAR agonist [160], there has been no investigations on its relationship with FATP4 expression in skeletal muscle. The transcriptional regulation of FATP4 in muscle cells is still elusive. However, muscle FATP4 expression is increased upon contraction by excercise [66,174] or electric-pulse stimulation [175] in mice. Similarly, the sensitivity of muscle FATP4 is enhanced by insulin [66,118]. FATP4 may thus facilitate insulin sensitivity for muscle FA β-oxidation. As muscle mass is regulated by myostatin, the deletion of this gene increases muscle FATP4 expression which leads to muscle hypertrophy together with reduction of body fat accumulation [176]. The deletion of a zinc transporter znt7 in mice also increases muscle FATP4 expression, and this results in lean phenotypes thus highlighting an indirect role of zinc on muscle FA β-oxidation [177]. During plamitate stress in C2C12 myotubes, vascular endothelial growth factor B treatment is shown to reduce lipid droplet accumulation concomitant with upregulation of FATP4 [178]. Hence, ample evidence supports the role of FATP4 in FA β-oxidation muscle. In contrast with FATP4, FATP1 in muscle appears to mediate lipid deposition and subsequent insulin resistance [179]. Thus, FATP4 may serve as a potential target for improving insulin resistance in muscle.

### FATP4 in adipose tissue

#### FATP4 role on lipid metabolism in adipose tissue

Adipose tissue is the main lipid storage of the body, and adipocytes control flux of FAs to peripheral tissues by storing and hydrolyzing TGs under hormonal controls [180,181]. It has been shown that FATP1 and FATP4 expression and FA-uptake activities are increased following insulin-induced differentiation of mouse 3T3-L1 fibroblasts to adipocytes in part by promoting membrane trafficking of intracellular FATP1 and FATP4 to the plasma membrane [47]. Moreover, TNF-α, which induces insulin resistance via tyrosine hypophosphorylation of insulin receptor [182], has been shown to inhibit adipocyte expression of FATP1 and FATP4 [183] and attenuate insulin-induced long-chain FA uptake in adipocytes [184]. Hence, FATP1 and FATP4 may be the major effectors in the regulation of FA flux into and out of adipocytes exerted by insulin [47,185]. These two enzymes may thus play an important role in energy homeostasis and metabolic disorders in adipose tissue. However, an opposite finding has been reported for endothelial cells. An increase in FATP4 expression was observed during TNF-α-stimulated endothelial autophagy and NF-κB signaling as well as palmitate-induced transcytosis across microvascular endothelial cells [186]. Thus, FATP4 is differentially regulated by TNF-α in adipocytes versus in endothelial cells.

In acquired obesity in humans, FATP4 expression is up-regulated in adipose tissues and closely correlated with increased body-mass-index, body-fat mass, and insulin resistance [82,83,147,187]. Genetic polymorphisms of FATP4 are associated with metabolic parameters in humans [38] as well as body-fat mass in pigs [188] and chickens [110]. FATP4 and FAT/CD36 expression as well as TG synthesis in adipose tissue of African-American women are higher than those of Caucasian women [147,187]. These findings suggest a significant role of FATP4 in adipose fat accumulation and metabolism in vertebrates.

An earlier study has shown that FATP4 knockdown in adipocytes has no effects on preadipocyte differentiation and FA influx but markedly increases basal lipolysis resulting in reduced cellular levels of TGs [67]. In the context of TG lipolysis, we have consistently shown that the levels of plasma glycerol and NEFA are further elevated in adipose-specific Fatp4 knockout (Fatp4A**^-/-^**) mice fed with methionine- and choline-deficient (MCD) diet [189] (Figure 3C). It is known that methionine- and choline-deficiency in cultured adipocytes and in mice fed with MCD diet triggers an induction of TG lipolysis detectable in culture medium and blood, respectively [149,150]. Consistently under *ex vivo* conditions, an exposure of mouse adipose tissues to MCD medium increased the secretion of glycerol and NEFA, and this secretion was further increased by adipose FATP4 deficiency [189]. Fatp4A**^-/-^** mice under chow diet already showed an increase in blood lipoproteins and leptin but a decrease in β-oxidation products, ketone bodies. These mutant mice when fed with MCD diet showed a further elevation of plasma leptin with suppression of plasma ketone bodies. Thus, ketone bodies may be regulated by leptin by adipocyte FATP4 deficiency *in vivo* (Figure 3C). The mechanism for an increase in leptin by the deficiency is not known and speculatively may involve an increase in malonyl-CoA [189]. Despite elevated blood glycerol and NEFA, an increase of leptin seen in MCD-fed mutants may in turn attenuate hepatic steatosis (Figure 3C). Despite steatosis protection, liver fibrosis is not protected. Thus, adipose FATP4 deficiency during MCD-induced non-obese MAFLD leads to metabolic disorders with elevated blood lipids in a similar manner as hepatocyte Fatp4 deficiency under high-sugar/high-fat conditions (Figure 3B,C).

In 2011, our first study on Fatp4A**^-/-^** mice was carried out in obese NAFLD model by HFD feeding for 12 weeks [190]. Upon intragastric loading of 9,10-^3^H labelled palmitate or 1-^14^C labelled lignocerate, there was no difference in flux activity in white adipose tissues between control and Fatp4A**^-/-^** mice. This indicates that adipose FATP4 does not play any role in FA uptake *in vivo*. Upon HFD feeding, Fatp4A**^-/-^** mice showed a significant increase in body weights, adipocyte expansion, and the development of fatty liver. Lipidomics analysis showed a decrease in subcutaneous fat PLs and SPLs concomitant with an increase in TGs. Thus, for adipocytes the shift of FA from PLs and SPLs towards TGs was already observed in 2011 (Figure 3D). Taken together, adipose Fatp4 deficiency elicits an opposing effect in suppressing and exacerbating hepatic steatosis in the non-obese MCD (Figure 3C) and obese HFD (Figure 3D) model, respectively.

Since aP2-Cre (or Fabp4-Cre) mouse line was used to generate Fatp4A**^-/-^** mice, it has been a concern whether these mutants could also exhibit FATP4 deletion in macrophages because aP2/Fabp4 is also highly expressed in macrophages [191]. Therefore, we generated macrophage-specific Fatp4-deficient mice (Fatp4M**^-/-^**) by cross-breeding LysM-Cre with Fatp4flox mouse line [192]. Bone marrow-derived macrophages (BMDMs) from control and Fatp4M**^-/-^** mice were utilized to investigate ER stress response *in vitro* [192]. Under basal levels, Fatp4M**^-/-^** BMDMs showed a decrease in cellular sphingomyelin and 24:1 ceramides indicating FATP4 role in the syntheses of SPLs and VLCFA-containing ceramides. These results are consistent with a previous report in keratinocytes [76,102]. ER stress in control BMDMs suppressed the levels of SPLs which were further suppressed by FATP4 deficiency. During ER stress, the deficiency also caused a further increase in cellular TGs. Thus, these results are again consistent with FA metabolic shift towards TGs as seen in FATP4-deficient enterocytes, hepatocytes, and adipocytes under dietary stress (Figure 3). Collectively, our data have demonstrated a fine balance between polar lipids (e.g*.*, PLs and SPLs) and neutral lipids (e.g*.*, TGs and CEs) regulated by FATP4 in intestine [123,127], liver [142,152,155], adipocytes [189,190], and macrophages [192]. Via TG metabolism, FATP4 deficiency causes worsen metabolic outcomes with elevated blood lipids and exaggerated fatty liver disease in diet-induced obese MAFLD/NAFLD (Figure 3).

Mechanistically, FATP4 localized in the ER of adipocytes is shown mediates FA uptake indirectly [60,61]. FATP4 is present only in detergent-soluble membranes but not in detergent-resistant membranes (lipid-raft domains) of adipocytes [193,194]. As FAT/CD36 localized at the plasma membrane is the main adipocyte FA transporter, FATP4 is not co-localized with FAT/CD36 nor directly interacts with lipid-raft associated FAT/CD36. This highlights the function of FATP4 in adipocytes as an intracellular ACS in mediating FA uptake by metabolic trapping and balancing polar and neutral lipids during dietary stress in non-obese (Figure 3C) and obese (Figure 3D) MAFLD/NAFLD models.

#### Transcriptional factors affecting FATP4 transcription in adipose tissue

Upon depletion of a histone demethylase, FATP4 expression in adipose tissues is decreased resulting in impaired adipogenesis [195]. These data suggest epigenetic mechanism for regulation of adipose FATP4. As biotin attenuates many parameters of metabolic syndrome disease, biotin supplementation is shown to up-regulate adipose FATP4 expression [196]. This up-regulation may result in suppression of adipose TG lipolysis [67] hence improving hyperlipidemia and metabolic syndrome. A flavonoid derivative Aaicalein has been developed as an activator of peroxisomal β-oxidation [197]; however, this compound does not have any effects on FATP4 expression in skin fibroblasts from X-ALD patients [198]. These data also support the notion that FATP4 is not the key enzyme but acts as a co-factor for VLCFA β-oxidation in X-ALD [90].

Here, we discuss the transcriptional factors that regulate adipocyte FATP4 transcription. It has been shown that FATP genes are regulated by PPARα and PPARγ in 3T3-L1 adipocytes [138]. In white adipose tissues of obese mice, PPARγ activators such as pioglitazone and troglitazone, have been shown to increase the expression of FATP genes more efficiently than PPARα activator clofibrate [199]. PPARγ ligand also induces FATP expression in 3T3-L1 adipocytes quicker than PPARα activator [173]. A correlation between PPARγ and FATP4/CD36 expression in human adipose tissues has also been reported [147]. Thus, PPARγ is the major regulator of FATP4 expression in human and mouse adipocytes (Figure 4). This is consistent with the observations in intestine in which PPARγ and FATP4 are highly expressed [112]. Thus, FATP4 is regulated by PPARs in hepatocytes [162] and adipocytes [173,199,200]. While PPARγ regulates FATP genes [138], knockdown of FATP4 in 3T3-L1 adipocytes on the other hand leads to an increase in PPARγ expression [67]. Knockdown of ACSL1 in human hepatocytes is also shown to increase PPARγ expression [201]. Similar to the events in enterocytes and hepatocytes, FATP4-mediated changes in FAs and fatty acyl-CoAs [141,157,158] could regulate PPARγ in a feedback-loop manner in adipocytes (Figure 4).

## Conclusions

FATP family members are a class of enzymes involved in FA activation and metabolism and they have significant contribution on the maintenance of lipid homeostasis. Among these members, FATP1 and FATP4 are the only two evolutionary conserved genes from invertebrates to vertebrates. FATP4 is the only member whose mutations or deletion can cause death in humans and animals. FATP4 was originally considered as an FA transporter, it has been now well accepted as an ACS. While the skin is the organ most affected by FATP4 mutations or deficiency, accumulating evidence has indicated a significant role of FATP4 in other metabolically active tissues, such as, intestine, liver, muscle, and adipose tissues. The evidence against FATP4 as an FA transporter has been published by several investigators including us. The up-regulation of FATP4 expression in adipose tissue with acquired obesity and in the liver with MAFLD/NAFLD indicates an involvment of FATP4 in these metabolic diseases. This is supported by association of FATP4 polymorphisms with body-mass index, body-fat thickness, and blood TGs. Our research work using tissue-specific Fatp4-deficient mice has demonstrated a significant role of FATP4 in cellular metabolism of polar and neutral lipids, and that its deficiency leads to a metabolic shift towards TGs, lipoproteins, and lipolysis products (Figure 3). Our research results highlight FATP4 as a model ACS that regulates lipid metabolism not only in skin but also metabolically active tissues. The worsen metabolic outcomes observed in tissue-specific Fatp4-deficient mice may be applicable to patients with FATP4 mutations who comsume high-sugar and/or high-fat diets.

## Abbreviations

ACS: acyl-CoA synthetase
ACSVL: very-long-chain acyl-CoA synthetase
ALDP: ATP-binding cassette half-transporter, adrenoleukodystrophy protein
BMDM: bone marrow-derived macrophages
CE: cholesterol ester
FA: fatty acid
FATP: fatty acid transport protein
HFD: high-fat diet
iPLA2β: group VIA calcium-independent phospholipase A2
IPS: ichthyosis premature syndrome
LCFA: long-chain fatty acid
LPC: lysophosphatidylcholine
LPCAT3: lysophosphatidylcholine acyltransferase3
MAFLD: metabolic (dysfunction) associated fatty liver disease
MCD: methionine-choline deficient
NAFLD: non-alcoholic fatty liver disease
NEFA: non-esterified free fatty acid
PC: phosphatidylcholine
PE: phosphatidylethanolamine
PL: phospholipid
PPAR: proxisome proliferator-activated receptor
PUFA: polyunsaturated fatty acid
SPL: sphingolipid
TG: triglyceride
VLCFA: very long-chain fatty acid
X-ALD: X-linked adrenoleukodystrophy
